# Measuring What Matters—A Holistic Approach to Measuring Well-Being in Endometriosis

**DOI:** 10.3389/fgwh.2021.770366

**Published:** 2021-12-21

**Authors:** Lori McPherson, Siladitya Bhattacharya

**Affiliations:** ^1^Department of Obstetrics and Gynaecology, NHS Grampian, Aberdeen, United Kingdom; ^2^Department of Obstetrics and Gynaecology, School of Medicine, Medical Sciences and Nutrition, University of Aberdeen, Aberdeen, United Kingdom

**Keywords:** endometriosis, quality of life (QOL), capabilities, gynaecology, women

## Abstract

Endometriosis is a common condition which affects women in a number of ways and impairs their ability to live a full and meaningful life. Evaluative research has traditionally taken its cue from a medical approach which has forced women to choose one area of functioning as their primary concern, and tended to use a narrow definition of treatment success which ignores general well-being. While recent trials have included quality of life (QOL) measures as outcomes, these have not been able to capture the totality of the impact of the disease and its treatment on a woman's capability to do what she might want to do and be who she might want to be. A capability approach might overcome this barrier, but the available tools will need to be refined and validated in women with endometriosis before this can be integrated within everyday clinical and research practice.

## Introduction

One in ten women of reproductive age lives with endometriosis. The proliferation of hormone sensitive endometrial cells outside the uterus leads to cyclic bleeding, inflammation, and scarring—mainly within the pelvis but also, occasionally, other parts of the body (1). The commonest symptoms of endometriosis are pelvic pain and infertility but the chronicity of the condition, side effects of treatment and the need for a surgical diagnosis can have a profound impact on many different aspects of a woman's life. The condition can progress over time and many women require multiple surgical procedures (2). The cornerstone of clinical management is symptom control and the conventional approach has been to dichotomise the physical symptoms of the disease into either pain or infertility before offering targeted treatment. Although many women present with both pain and infertility, they are often advised to prioritise one of these as hormonal treatments for pain can interfere with ovulation and are incompatible with attempts to conceive.

Randomised trials assessing the effectiveness of treatments for endometriosis have tended to respect this artificial division and focused on live-birth or severity of pain as outcomes. More recently, the importance of measuring QOL as a global indicator of treatment success has been recognised, but current methods for assessing QOL in endometriosis have been inconsistent and have not been designed to capture the totality of the impact of the condition on women's well-being (3, 4).

In this article we review outcomes reported in randomised trials of treatments for endometriosis and tools used to measure these. We also consider an alternative choice driven capability-based approach (5, 6). which aims to shift the focus from measuring utility (happiness) to evaluating functionings and capabilities in women (7).

## Outcomes and Tools Used in RANDOMISED Clinical Trials

We undertook a computerised search of the Cochrane Library in order to identify reviews of randomised trials of treatments in endometriosis. From an initial list of 52 eligible articles, 3 were excluded as they (3) had been withdrawn from the Cochrane Library. Of the remaining 49, 29 were excluded after screening the abstracts as they did not conform to our inclusion criteria, leaving 20 articles for final analysis (Appendix 1). Fertility was the chosen primary outcome in 4 reviews, pain in 12 and both in 4. In the reviews with pain as the primary outcome, fertility was considered a secondary outcome in 3; of the 4 reviews with fertility as a primary outcome, 1 review included pain as a secondary outcome.

Of the reviews focusing on fertility, 6 selected live birth rate as the outcome of interest (8–13). Hart et al. (14) reported clinical pregnancy rate and Brown and Farquhar (15) included all pregnancy outcomes; including live birth, miscarriage, and ectopic pregnancy etc. In actual fact, due to the nature of the data in the primary trials, only 3 of the Cochrane reviews were actually able to report live births, while the others reported rates of clinical pregnancy or viable pregnancy. In three reviews, all participants were undergoing fertility treatment (IVF or ICSI) (8, 10, 11). Hughes et al. (9) included participants who were deemed infertile, having failed to conceive after 12 months of unprotected intercourse. While all 14 randomised control trials (RCTs) included in the systematic review by Balfort et al. (12) commented on fertility outcomes, only 3 included women with a diagnosis of infertility. Similarly, the review by Lu et al. (13) included 4 RCTs of which 3 focused on participants with a confirmed diagnosis of infertility. None of the 3 RCTs reviewed by Hart et al. (14) specifically included participants with infertility.

Pain was a key outcome for many of the reviews and the most common tool used to assess it was the visual analogue scale (VAS) which figured in 11 reviews (12–23). Eight reviews used dichotomous outcomes (e.g., improved or not improved) to assess pain (14–16, 18, 19, 21, 23, 24). The Biberoglu and Behrman rating scale was used by both Lu et al. (22) and Brown and Farquhar (15). This was modified to take into account the impact on work and loss of function by Abou-Setta et al. (17), who also used the Total Endometriosis Severity Profile (TESP) to assess pain. The Endometriosis Symptoms Severity Score (ESSS) was used by 2 reviews to assess pain (15, 23). Two reviews used a specific set of Chinese validated outcomes to assess pain (15, 25). Flower et al. (26) and Brown and Farquhar (15) assessed pain using the 15-point Guideline for Clinical Research on New Chinese Medicine for Treatment of Pelvic Endometriosis scale, while Brown et al. (20) used the verbally reported scale (VRS). One review used an unspecified “pain questionnaire” to assess pain (27).

Three reviews reported recurrence of disease, as assessed at repeat laparoscopy using the standard or revised American Fertility Society (AFS) (18, 19, 24), revised American Society for Reproductive Medicine (ASRM) score or Endoscopic Endometriosis Classification (EEC) score (19) as their primary outcome.

Seven reviews also reported on adverse outcomes, complication rate or side effects of treatment (10, 11, 15, 16, 21, 22, 24). Farquhar et al. (24) was the only review to select patient satisfaction with treatment. Although none of the reviews reported on QOL as a primary outcome, 7 aimed to assess this as a secondary outcome measure. Only 3 were able to report data on QOL. Fu et al. (21) and Zhu et al. (25) used the short form 36 (SF-36) as a QOL instrument. Balfort et al. (12) used the European Quality of life 5 dimension (EQ-5D) index summary, the short form 12 (SF-12) and the Endometriosis Health Profile-30 (EHP-30).

These data confirm the traditional emphasis on pain and fertility, rather than QOL as primary outcomes in those undertaking randomised trials in endometriosis as well as those engaged in evidence synthesis. Although some systematic reviews have included QOL as a secondary outcome, the variety of tools used (not all of which are disease-specific) make the results difficult to interpret. Yet, endometriosis is a multidimensional disease which does not just affect a woman physically but also causes anxiety and depression, compromises social relationships (28) and affects opportunities for education, employment and reproduction. QOL measures have sought to capture general well-being in women with endometriosis using a variety of techniques but, as yet, there is no consensus on the best way of achieving this (4). The most commonly used tools were SF-36 and the EHP-30. SF-36 is a generic QOL instrument which has been validated for use in endometriosis. Although it allows comparison between different diseases and the general population, it is likely to miss certain unique aspects of endometriosis such as dyspareunia, often described as a debilitating symptom. EHP-30 is a disease-specific tool for endometriosis, but while it examines 11 different domains, it does not provide a single score for QOL, thus limiting its appeal amongst researchers seeking a single outcome for a clinical study (29).

This review included studies conducted mainly in medium and high income countries with the findings written in English. As such, it may not be entirely representative of women across the world especially those from low income countries. This is a limitation of the review when considering its implications in a global context.

## A New Core Outcome Set for Clinical Trials

Recently, a multidisciplinary international group, including women living with endometriosis, has used robust methods to standardise outcome selection, collection and reporting across randomised trials and systematic reviews in endometriosis (4). Three core outcomes for trials evaluating potential treatments for pain and other symptoms associated with endometriosis include overall pain, improvement in the most troublesome symptom and QOL. In trials evaluating potential treatments for infertility associated with endometriosis, 8 core outcomes were identified. These include viable intrauterine pregnancy confirmed by ultrasound, pregnancy loss (including ectopic pregnancy, miscarriage, stillbirth, and termination of pregnancy), live birth, time to pregnancy leading to live birth, gestational age at delivery, birthweight, neonatal mortality, and major congenital abnormalities. Adverse events and patient satisfaction with treatment were identified as core outcomes applicable to all trials (4).

## Are We Measuring What Matters?

The core outcome set includes QOL in women with pain, but not infertility associated with endometriosis. While endometriosis is associated with psychological morbidity and reduced QOL, mainly due to the impact of chronic pelvic pain (3), the experience of childlessness can affect well-being (30). A systematic review (3) has highlighted the paucity of studies using QOL as an outcome, along with a lack of long term follow up studies and methodological issues in the design of QOL instruments, especially as generic tools have limitations in terms of measuring chronic conditions which fluctuate over time. Despite their obvious advantages, some of the common disease-specific questionnaires have been found to have design problems, and it has been suggested that it might be prudent to use more than one type of tool (3).

Standard QOL tools such as SF-36 and EQ-5D can provide an estimate of the effect of endometriosis on day-to-day functioning and provide health funders and policy makers with quality adjusted life years (QALY) for comparisons with other health conditions in order to inform health policy. Yet QALYs are hardly ever used to inform decision making at a personal level and patients can (and do) have the right to refuse treatments on grounds of personal autonomy. Thus, these generic QOL measures fall short of being able to describe the totality of a woman's experience, provide a comprehensive estimate of the full impact of an intervention on a woman's well-being or act as a practical guide to joint clinical decision making in everyday practice.

Disease-specific tools can capture the impact of endometriosis on different aspects of a woman's life but cannot fully incorporate a woman's inability to realize her reproductive and other life plans. This is problematic, as any health gain in a narrow physical domain could come at an unacceptably high cost in terms of unfulfilled expectations and absent opportunities. A woman who chooses not to have children might have the same fertility outcome as someone who is unable to have children either due to endometriosis or the effects of its treatment, but the two have very different states of well-being.

The capabilities approach developed by Amartya Sen (7) and Nussbaum (31) allows clinicians and researchers to think more globally in terms of measuring the impact of a condition and its treatment, not just on their immediate physical state but their ability to lead a life which has value. Capabilities represent functions which are essential to a flourishing life or “eudaimonia” (5), including attributes such as normal life span; bodily health; bodily integrity; senses; imagination and thought; emotions; practical reason; affiliation; the ability to appreciate and interact with other species; play and control over one's environment (32). Functionings are things a person can do (exercise, work, play) or achieve (employment, parenthood) (33) but measurement of achieved functionings may be insufficient for a complete assessment of well-being which should also include a person's freedom to achieve their goals in life (34). Thus, measuring achievement (ability to perform physical and mental tasks) is less meaningful unless considered alongside capabilities (34). This approach, which accepts the multidimensional nature of well-being and recognises the importance of choice, represents a more holistic method of reviewing health in endometriosis; which not only limits what women can do in terms of everyday tasks, but also what they might plan to achieve in their lives (35).

## Existing Capability Measures

A number of research groups have attempted to develop capability-based measures which currently include tools such as the Oxford Capability instrument (OxCap) (36, 37), the Adult Social Care Outcome Toolkit (ASCOT) (38, 39) and the ICEpop CAPability measure (ICECAP) (40, 41). OxCap (Oxford Capability) comprises a 64 item questionnaire based on analysis of secondary data from household and panel surveys and has since been adapted to different settings (37, 42). ASCOT was developed to measure capability specifically in relation to social care and has since evolved through four versions, with Sen's capability theory appearing only relatively late in its development (38, 43). ICECAP (40) was initially targeted at older people but subsequent work has been done to develop the five-attribute ICECAP-A measure for the adult population (41) and the seven-attribute ICECAPSCM, a supportive care measure for use with people at the end of life (44). These tools have varied in their treatment of the issue of capabilities vs. functionings as well as their basis for the capabilities evaluated. Whilst the identification of attributes for the OxCap has been expert led, the ICECAP and ASCOT instruments were developed through participatory methods involving qualitative work with representative groups. Nussbaum's concept of central capabilities (31) has influenced the approach used by Anand et al. (36), who used questions from the British Household Panel Survey to assess capability and refine the later versions of OxCap. In contrast, ASCOT and ICECAP started with the premise that it was important to venture beyond health, with the move to capability coming later. For ICECAP researchers, the impetus for a capability approach came from results from qualitative research (40).

All current approaches are still at a relatively early stage but there is increasing evidence of the use of these measures in research (45). Areas for further development have been identified; such as the use of different approaches to generate both measurement systems and their valuation, exploration of their sensitivity over the life course (46) and ways of using the capability approach in decision making. A fundamental problem is the fact that a measure of capability cannot be generated from questions about the ability to undertake actions (33). In addition, measuring an individual's entire capability set is challenging and cannot just be based on the totality of functionings (33). There have been some initial suggestions about how these issues can be addressed, but it is clear that much more work is needed.

## Conclusion

Endometriosis is a common condition which affects women in a number of ways and impairs their ability to live a full and meaningful life. Evaluative research has traditionally taken its cue from a medical approach which has forced women to choose one area of functioning as their primary concern and tended to use a narrow definition of treatment success which ignores general well-being. While recent trials have included QOL measures as outcomes, these have not been able to capture the totality of the impact of the disease and its treatment on a woman's capability to do what she might want to do and be who she might want to be. The multidisciplinary implementation of a capability approach might overcome this barrier, but the available tools will need to be refined and validated in women with endometriosis before this can be integrated within everyday clinical and research practice.

## Author Contributions

LM drafted the manuscript after conducting a review of the literature. SB edited the manuscript and expanded it. Both authors contributed to the article and approved the submitted version.

## Conflict of Interest

The authors declare that the research was conducted in the absence of any commercial or financial relationships that could be construed as a potential conflict of interest.

## Publisher's Note

All claims expressed in this article are solely those of the authors and do not necessarily represent those of their affiliated organizations, or those of the publisher, the editors and the reviewers. Any product that may be evaluated in this article, or claim that may be made by its manufacturer, is not guaranteed or endorsed by the publisher.

## Figures and Tables

**Figure 1:**
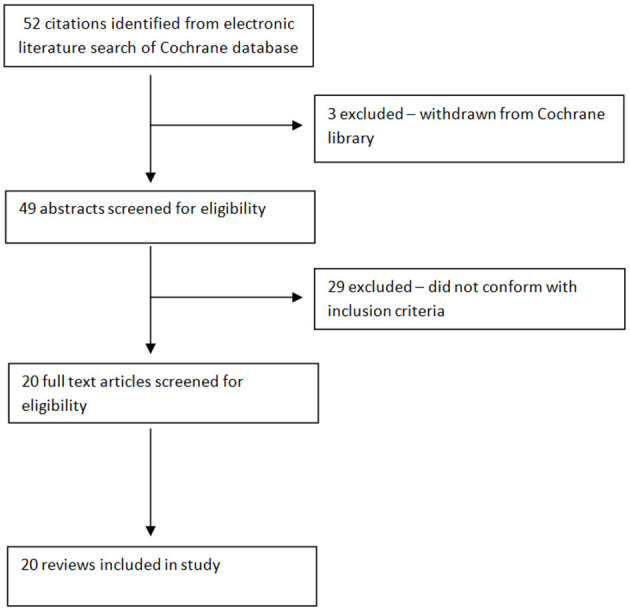
Study flow diagram.
